# Challenging case of leadless pacemaker implantation for a patient with left-sided inferior vena cava

**DOI:** 10.1016/j.hrcr.2026.07.005

**Published:** 2026-07-15

**Authors:** Yuki Tanaka, Masaru Yamaki, Yoshifumi Mizuguchi, Yasumi Igarashi

**Affiliations:** Department of Cardiology, Sapporo Kosei General Hospital, Chuo-ku, Sapporo City, Hokkaido, Japan

**Keywords:** Leadless pacemaker, Anomaly, Left-sided IVC, α-Loop advancement, Complex venous access

**Key Teaching Points**

• This is the first case report of successful leadless pacemaker implantation via the left-sided inferior vena cava (IVC) from the femoral vein.
• The challenges of leadless pacemaker implantation via the left-sided IVC from the right femoral vein access included poor catheter manipulation owing to friction caused by catheter bending, along with the sigmoid curve at the level of the renal veins, and difficulty in directing the catheter to the right ventricular septum because of advancing from the IVC to the right atrium in the opposite direction to the right ventricle.
• The α-loop advancement of the catheter, partial catheter withdrawal to reduce its bending, and covering the catheter with the sheath to improve torque transmission overcome the challenges of Aveir VR implantation via left-sided IVC.

## Introduction

A left-sided inferior vena cava (IVC) is 1 of the anatomical variants of IVC. The prevalence of left-sided IVC in the population ranges from 0.1% to 0.4%.^1^^,^^2^ Several studies have reported successful catheter ablation from right femoral access via the left-sided IVC without complications.^3^^,^^4^ However, to our knowledge, there are no case reports of successful implantation of a leadless pacemaker (LP) via a left-sided IVC.

## Case report

A 69-year-old man with chronic myeloid leukemia was hospitalized in the hematology department of our institution for the treatment of generalized edema as a side effect of dasatinib. Electrocardiographic monitoring showed frequent sinus pauses for 3 seconds at a time. He was asymptomatic, but the cardiac arrest did not improve after withdrawal of dasatinib and bisoprolol. Considering his risk for infection, we decided to implant an LP.

His body mass index was 24.6 kg/m^2^. He had a history of chronic heart failure, paroxysmal atrial fibrillation, and chronic obstructive pulmonary disease. Blood examination showed a B-type natriuretic peptide level of 197.4 pg/mL, white blood cell count of 4900/μL, hemoglobin of 8.3 g/dL, and platelet count of 22.3 × 10^4^ /μL. Chest X-ray imaging revealed a cardiothoracic ratio of 54%, without congestion and effusion. Electrocardiography revealed sinus rhythm without conduction block. Echocardiography showed a left ventricular ejection fraction of 50% without wall motion asynergy, left atrial volume index of 45.6 mL/m^2^, and trivial tricuspid regurgitation and trivial aortic regurgitation. Contrast-enhanced computed tomography (CT) showed that the right common iliac vein crossed to the left side posterior to the left common iliac artery, the left-sided IVC crossed to the right side anterior to the aorta at the level of the renal vein, and finally flowed into the right atrium (RA) (Figure 1A and 1B). This was a typical finding of left-sided IVC. However, the anomaly was not recognized during the preprocedural assessment and was identified only after the procedure.

**Figure 1:**
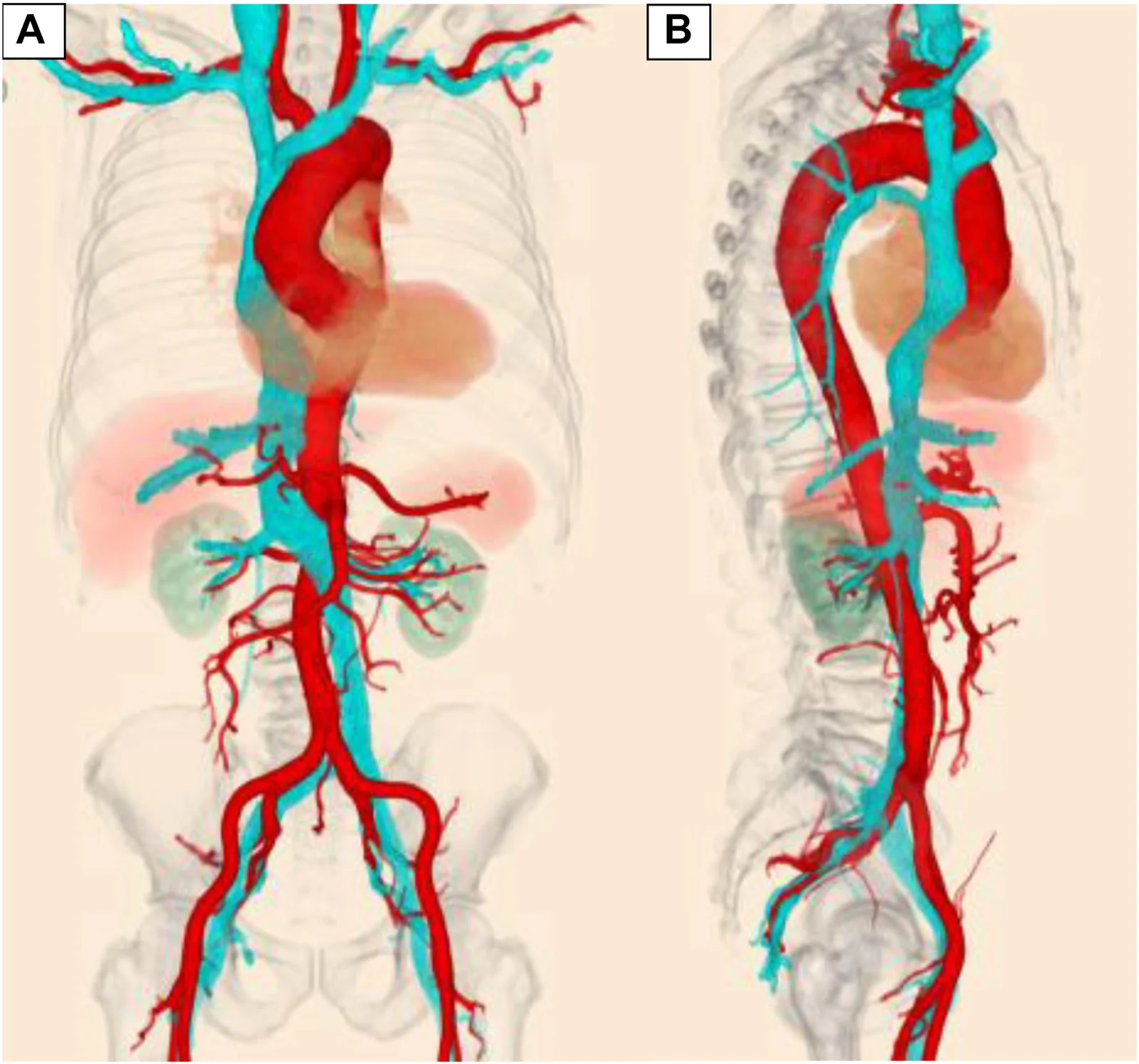
**A:** Contrast-enhanced cardiac computed tomography (CT) image of the anterior–posterior view showing left-sided inferior vena cava (IVC). Veins are shown as *blue lines*. Arteries are shown as *red lines*. The right common iliac vein crossed to the left side posterior to the left common iliac artery, the left-sided IVC crossed to the right side anterior to the aorta at the level of the renal vein, and finally flowed into the right atrium (RA). **B:** Contrast-enhanced cardiac CT image of the right lateral view. AP = anterior–posterior; CT = computed tomography; IVC = inferior vena cava; RA = right atrium.

The procedure was started using the right femoral vein approach. A guide wire was advanced to the left side of the spine and brought back to the right side just before advancing to the RA. 8-Fr and 16-Fr short sheaths (Medikit Co., Ltd., Tokyo, Japan) were then inserted into the right femoral vein for dilatation of the puncture point. A 5-Fr pig-tail catheter (Technowood, Tokyo, Japan) was advanced into the right ventricle (RV) for right ventriculography. When the catheter was advanced into the RA from the IVC, the catheter was directed toward the RA posterior wall on the right anterior oblique (RAO) view (Figure 2A). A 25-Fr sheath was then advanced into the IVC using an Amplatz Super Stiff wire (Boston Scientific, Marlborough, MA) smoothly and set at the IVC directly below the RA. Aveir VR (Abbott, Chicago, IL) was advanced into the RV; however, the catheter could not be directed toward anything other than the right ventricular lateral wall by manipulating the deflection lever of the catheter and catheter rotation (Figure 2B). Aveir VR could be advanced toward the right ventricular septum with α-loop advancement along the lateral wall of the RA and RV (Figure 2C). We attempted to screw the Aveir VR into the right ventricular septum after confirmation of appropriate impedance, sensitivity, and threshold. However, rotational manipulation of the LP control knob failed to transmit rotational force to the Aveir VR (Figure 2D). Therefore, we tried to reduce the pronounced bending of the catheter by withdrawing the catheter with clockwise torque force, keeping contact between the Aveir VR and right ventricular septum. We re-attempted to screw the Aveir VR into place; however, the Aveir VR could rotate only about halfway round, owing to the poor transmission of rotational manipulation (Figure 2E). After retrieving the Aveir VR, we recreated the conditions before screwing it in. Subsequently, we tried to cover the catheter with the sheath to improve torque transmission of the rotational manipulation. Finally, we screwed the Aveir VR into the right ventricular septum slowly but completely (Figure 2F). The procedure was finished with a pacing threshold of 0.25 V/0.4 ms, sensitivity of 15.3 mV, and impedance of 1040 Ω. We did not require an additional sheath, such as that used for a snaring technique, and achieved Aveir VR implantation using only the standard delivery sheath. The patient was discharged from our institution without complication. No incidence of pacemaker-related complication was observed for 6 months following discharge.

**Figure 2:**
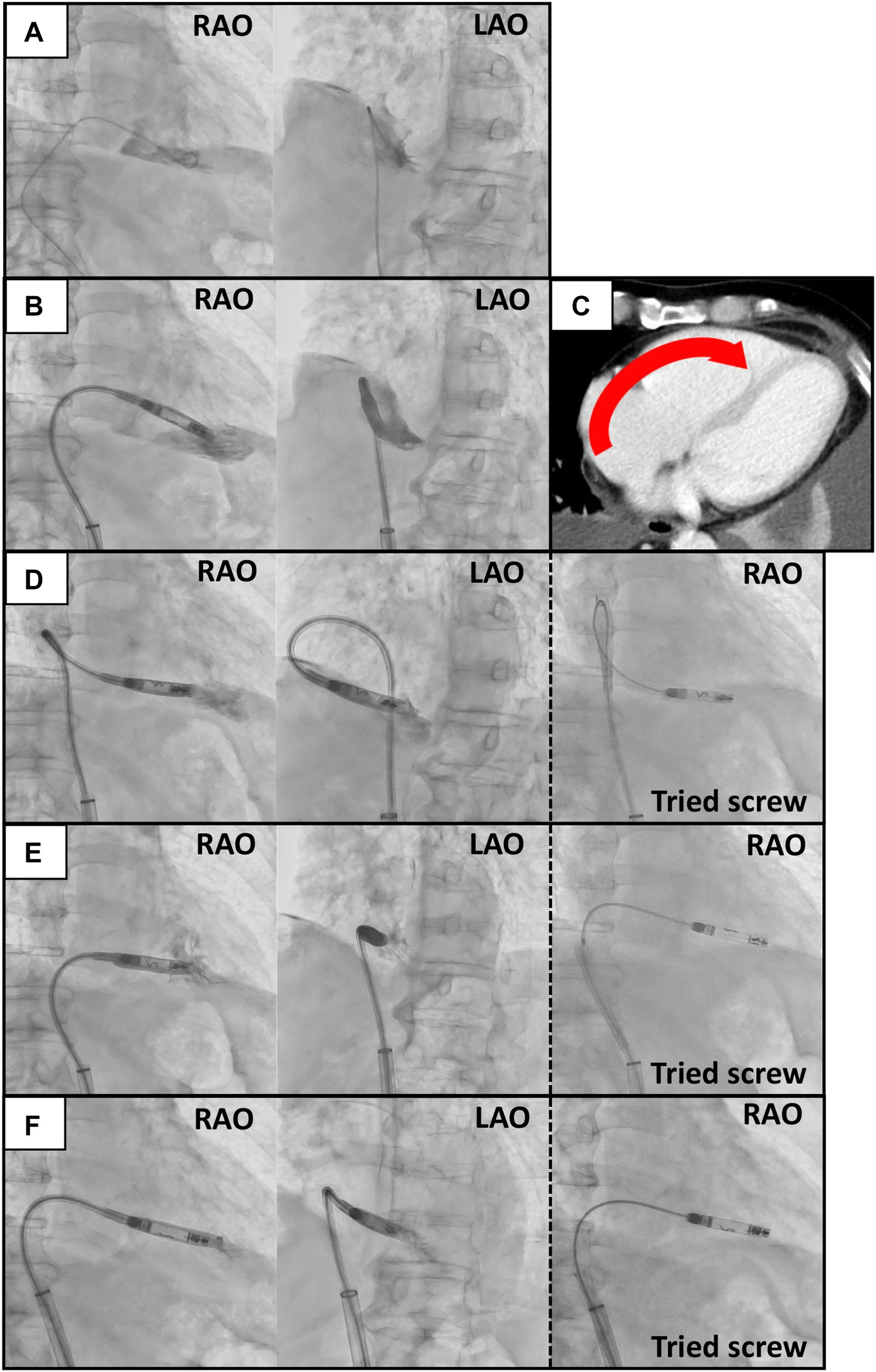
**A:** Right ventriculography in the right and left anterior oblique views. The catheter was advanced into the RA from the IVC, directed toward the RA posterior wall. **B:** Fluoroscopic images obtained during the first attempt. The tip of the catheter was positioned against the right ventricular lateral wall. **C:** Contrast-enhanced CT cross-sectional image. The *red arrow* indicates the route of α-loop advancement of the catheter. **D:** Fluoroscopic images obtained during the second attempt after α-loop advancement of the catheter. Images to the left and right side of the *dotted line* show the catheter position before and after the screwing attempt, respectively. The tip of the catheter was positioned against the right ventricular septum. Aveir VR could not be screwed into the myocardium. **E:** Fluoroscopic images obtained during the third attempt after α-loop advancement followed by pulling back the catheter. Images to the left and right side of the dotted line show the catheter position before and after the screwing attempt, respectively. The catheter bending reduced after pullback. However, Aveir VR could rotate only approximately one-half turn. **F:** Fluoroscopic images obtained during the fourth attempt after α-loop advancement, catheter pullback, followed by covering the catheter with the sheath. Images to the left and right of the *dotted line* show the catheter position before and after the screwing attempt, respectively. Aveir VR was gradually rotated approximately 1 and a half turns. CT = computed tomography; IVC = inferior vena cava; LAO = left anterior oblique; RA = right atrium; RAO = right anterior oblique.

## Discussion

IVC develops through a process involving formation, regression, and anastomoses of 3 parallel pairs of embryonic veins (post-cardinal, sub-cardinal, and supra-cardinal veins). The final product of the remaining parts of these sets of veins becomes the adult IVC and iliac bifurcation.^5^ Left-sided IVC results from the regression of the right supra-cardinal vein with persistence of the left supra-cardinal vein, which is 1 of the last veins to disappear. The left IVC crosses over to the right side via the left renal vein in the majority of cases. This crossover is usually anterior to the aorta but rarely posterior.^6^ When the transcatheter cardiac procedure is performed via left-sided IVC, the sigmoid curve before and after passing the renal veins provides some challenges, such as the risk of venous injury and difficulty of catheter manipulation due to the friction caused by catheter bending. However, successful venous cannulation has been reported in patients with a left-sided IVC using a 23-Fr single cannula advanced to the superior vena cava (SVC) from the right femoral vein for minimally invasive cardiac surgery–aortic valve replacement without vascular complications and successful catheter ablation via the right femoral vein for atrioventricular nodal re-entry tachycardia or orthodromic atrioventricular reciprocating tachycardia involving a right posterior accessory pathway.^3^^,^^4^^,^^7^

The challenges of the present case were not only poor transmission of torque due to sigmoid bending through the renal vein but also the difficulty of advancing Aveir VR toward the right ventricular septum due to insertion into the RA opposite the RV. Aveir VR could not be directed to the right ventricular septum by manipulating the deflection lever of the catheter and catheter rotation. The α-loop advancement of Aveir VR solved the problem, but made it difficult to screw Aveir VR into the right ventricular septum owing to poorer transmission of the rotational manipulation of the LP control knob caused by pronounced bending of the catheter. Therefore, to solve this problem, we withdrew the catheter to decrease catheter bending and covered the catheter with the sheath to improve torque transmission. In retrospect, we considered several alternative strategies that might have facilitated LP implantation. If the left femoral approach had been selected, catheter bending may have been reduced, which might have improved catheter manipulation and torque transmission. If the Micra VR (Medtronic Inc., Minneapolis, MN) device had been chosen for LP implantation, optimization of torque transmission for pacemaker device fixation might not have been necessary, as only delivery of the device to the right ventricular septum would have been required. However, the Micra device cap might have been too rigid to be safely advanced in α-loop configuration because of the potential risk of cardiac perforation. Therefore, advancing the Micra system toward the right ventricular septum might have been challenging. We have summarized the differences between the 2 LPs to assist in procedural planning for anatomically challenging cases (Table 1).

**Table 1:** Differences between 2 leadless pacemakers

	Aveir	Micra
Implantation	Screw	Tines
Pre-mapping	Possible	Impossible
Atrioventricular synchrony	2 devices are needed (atrium and ventricle)	1 device is needed (ventricle)
Outer diameter of the sheath	27 Fr	27 Fr
Device sleeve/cup	Soft	Hard
Catheter shaft	More flexible	More supportive
RIJV approach cases	Reference no. 8	Reference nos. 9,10

RIJV = right internal jugular vein.

The efficacy and safety of Micra implantation via the right internal jugular vein (RIJV) were reported.^9^ This approach was often more effective in cases of infracardiac vein anomalies, such as the left-sided IVC in our patient. In a previously reported case of partial absence of the IVC, Micra VR implantation was performed by changing the approach site from the right femoral vein to the right jugular vein.^10^ In this case, a stiff guidewire advanced from the right femoral vein followed a tortuous route, crossing to the left side of the spinal column and reaching the SVC, but it could not be advanced into the IVC and RA. Therefore, the operator suspected an IVC anomaly, changed the approach site to the right jugular vein, and successfully implanted the Micra VR via the SVC. In our case, once the guidewire reached the RA through a tortuous route, changing the approach site to the right jugular vein could also have been considered. However, the feasibility of LP implantation via jugular vein access was not considered at that time, and the venous anomaly was not fully appreciated during the preprocedural assessment. Therefore, Aveir VR implantation was continued via the femoral vein. RIJV approach of Aveir VR was also reported, although in only a few cases.^8^ Therefore, Aveir VR implantation from the RIJV was 1 of the effective options in the present case. However, the RIJV approach may be associated with a higher risk of RV perforation because of stronger direct force transmission during the jugular approach than during the femoral approach, particularly in an apical position.^9^ Moreover, the RIJV approach cannot be chosen in some cases because of SVC occlusion owing to tumor or thrombus, mergers of SVC anomaly such as persistent left SVC, or the impossibility of device passage owing to previous multiple lead implantation. Therefore, our case of Aveir VR implantation via the right femoral vein using α-loop delivery configuration, followed by reduction of catheter bending, and sheath-assisted stabilization may represent a useful technique to overcome the challenges of Aveir VR implantation in patients with complex vessel anatomy, such as left-sided IVC.

## Conclusion

Left-sided IVC presents challenges to LP implantation. When the right femoral approach is selected, difficulties arise in directing the catheter toward the septum and in achieving adequate catheter torque transmission. The α-loop advancement technique, partial catheter withdrawal to reduce excessive bending of the catheter, and sheath-assisted stabilization to improve torque transmission enabled successful implantation of Aveir VR in such a challenging case as this left-sided IVC.

## Disclosures

The authors have no conflicts of interest to disclose.
